# Knockdown of lncRNA SNHG16 suppresses multiple myeloma cell proliferation by sponging miR-342-3p

**DOI:** 10.1186/s12935-020-1118-1

**Published:** 2020-02-03

**Authors:** Xi Yang, Hongming Huang, Xinfeng Wang, Haiyan Liu, Hong Liu, Zenghua Lin

**Affiliations:** 1 Department of Hematology, The First Affiliated Hospital of Soochow University, Suzhou, 215006 Jiangsu People’s Republic of China; 2 Department of Hematology, Affiliated Hospital of NanTong University, No.20 Xishi Road, Nantong, 226001 Jiangsu People’s Republic of China

**Keywords:** Multiple myeloma, Proliferation, lncRNA SNHG16, miR-342-3p

## Abstract

**Background:**

Aberrant expression of long non-coding RNAs (lncRNAs) is closely associated with development and prognosis of human cancers. LncRNA SNHG16 is reportedly involved in human cancer; however, its roles in multiple myeloma (MM) remain unclear.

**Methods:**

In this study, we investigated the function and molecular mechanisms of SNHG16 in MM. MM cells were transfected with si-SNHG16 or si-NC. SNHG16 expression levels was measured by qRT-PCR. Cell proliferation was monitored using the MTS. Flow cytometry assay was performed to measure the cell cycle and apoptosis. Luciferase reporter assay were performed to confirm the sponged miRNAs of SNHG16.

**Results:**

SNHG16 expression was up-regulated in MM tissues. SNHG16 knockdown suppressed cell proliferation, arrested cell cycle transition from G1 to S phase, and promoted the apoptosis of MM cells. Moreover, SNHG16 knockdown promoted cleaved-Caspase-3, cleaved-Caspase-9, Foxa3a, and Bax expression, while markedly inhibiting *CCND1*, Bcl-2, Cyclin D1, PI3K, and p-AKT expression in MM cells. miR-342-3p was a direct target of SNHG16. SNHG16 knockdown significantly increased miR-342-3p expression in MM cells. Overexpression miR-342-3p markedly suppressed cell proliferation, arrested cell cycle transition from G1 to S phase, and promoted apoptosis of MM cells. Overexpression of miR-342-3p markedly promoted cleaved-Caspase-3/-9, Foxa3a, and Bax expression, and inhibited *CCND1*, Bcl-2, Cyclin D1, PI3K, and p-AKT expression in MM cells. Additionally, repression of miR-342-3p could rescue the effect of SNHG16 knockdown on MM cell proliferation, cycle arrest, apoptosis, and related protein expression.

**Conclusion:**

Knockdown of lncRNA SNHG16 suppresses MM cell proliferation by sponging miR-342-3p, implicating SNHG16 as a novel therapeutic target for MM.

## Background

Multiple myeloma (MM) is a fatal plasmocyte malignancy [1]. MM is the most common hematological cancer worldwide, and its incidence has continued to rise annually [2]. Despite advances in the diagnosis and treatment for MM, including chemotherapy, autologous/allogeneic stem cell transplantation, and monoclonal antibodies therapy such as daratumumab, elotuzumab, indatuximab, SAR650984, the results of clinical treatment of MM remain unsatisfactory [3, 4]. MM pathogenesis is very complex and the detailed underlying mechanisms for the development and progression of MM remain largely unknown.

Increasing evidence indicates that the aberrant expression of long non-coding RNAs (lncRNAs) are closely associated with the development and prognosis of various types of cancer, including MM [5]. Liu et al. [6] reported that lncRNA TUG1 were significantly up-regulated in MM samples and cell lines, and that down-regulation of TUG1 markedly inhibited MM cell proliferation and promoted apoptosis. LncRNA Small Nucleolar RNA Host Gene 16 (SNHG16), an SNHG member, is up-regulated and functions as an oncogene in pancreatic cancer [7] and gastric cancer [8]. Although SNHG16 plays important roles in different cancers, its functional role and underlying molecular mechanism in MM tumorigenesis are still largely unclear.

LncRNAs act as competing endogenous RNAs (ceRNAs) to sponge microRNAs (miRNAs). Aberrant expression of miRNAs play critical roles in multiple biological processes in cancer, including MM [9]. miR-342-3p, which is localized to chromosome 14q32, is a tumor suppressor miRNA involved in non-small cell lung cancer [10] and osteosarcoma [11]. Given the suppressive role of miR-342-3p in cancer, we aimed to determine whether SNHG16 acts as an miR-342-3p sponge to regulate the proliferation and apoptosis of MM cells.

In this investigation, we first evaluated the expression of SNHG16 in MM samples cell lines. Subsequently, we explored the effects of SNHG16 on MM cell proliferation, cycle and apoptosis. Finally, the interaction between SNHG16 and miR-342-3p and the underlying mechanisms of SNHG16 in MM cells were investigated.

## Methods

### Clinical specimen collection

Twenty primary (MM patients 66.30 ± 8.21; male, 15) and 15 marrow healthy samples (control, age, 59.6 ± 11.89; male, 10) were collected from June 2018 to January 2019 at Affiliated Hospital of NanTong University. No treatment was applied before the sample collection. MM specimens were taken from intramedullary regions. This study obtained approval from the Clinical Research Ethics Committee of Affiliated Hospital of NanTong University. Informed written consent for the use of the tissue samples was obtained from all patients and healthy controls. All fresh tissues were frozen in liquid nitrogen immediately and stored at − 80 °C until use.

### Cell culture and transfection

Two human MM cell lines (RPMI-8226 and NCI-H929) were purchased from the Cell Bank of the Chinese Academy of Sciences (Shanghai, China). MM cell lines were cultured in RPMI-1640 (Gibco, Carlsbad, CA, USA) supplemented with 10% fetal bovine serum (FBS; Invitrogen, Carlsbad, CA, USA) and 1% penicillin–streptomycin (Sigma-Aldrich, St. Louis, MO, USA) at 37 °C in a humidified atmosphere of 5% CO_2_. MiR-342-3p mimics, miR-342-3p inhibitor, miR-negative control (miR-NC), siRNAs to SNHG16 (si-SNHG16), and si-NC were designed and commercially constructed by Genechem (Shanghai, China). All transfections were conducted using Lipofectamine 2000 (Invitrogen). The sequences used were as follows: si-SNHG16, 5′-GGAACAUACUGCUAUCAUAGA-3ʹ; si-NC, 5′-UUCUCCGAACGUGUCACGUTT-3ʹ; miR-342-3p mimics: 5′-UCUCACACAGAAAUCGCACCCGU-3ʹ; miR-NC: 5′-UUCUCCGAACGUGUCACGUTT-3ʹ; miR-342-3p inhibitor: 5′-ACGGGUGCGAUUUCUGUGUGAGA-3ʹ; NC inhibitor: 5′-CAGUACUUUUGUGUAGUACAA-3ʹ.

### Quantitative real-time PCR (qRT-PCR)

Total RNA was extracted from MM tissues or cell lines using TRIzol reagent (Invitrogen). For SNHG16, first strand cDNA was reverse transcribed from total RNA using the PrimeScript™ II 1st Strand cDNA Synthesis Kit (TaKaRa Bio, Dalian, China). For miR-342-3p, qRT-PCR was performed using TaqMan miRNA assays (Applied Biosystems, Foster City, CA, USA). All PCRs were performed using an ABI 7500 RT-PCR system (Applied Biosystems) with SYBR^®^ Premix Ex Taq™ Kit (TaKaRa Bio). PCR primers were purchased from GenePharma (Shanghai, China) with the following sequences: *CCND1* forward, 5ʹ-ATCAAGTGTGACCCGGACTG-3ʹ and reverse, 5ʹ- CTTGGGGTCCATGTTCTGCT-3ʹ.

SNHG16 forward, 5ʹ-CCTCTAGTAGCCACGGTGTG-3ʹ and reverse, 5ʹ-GGCTGTGCTGATCCCATCTG-3ʹ; 18srRNA forward, 5ʹ-CCTGGATACCGCAGCTAGGA-3ʹ and reverse, 5′-GCGGCGCAATACGAATGCCCC-3ʹ; miR-342-3p forward, 5ʹ- ACACTCCAGCTGGGTCTCACACAGAAATCGC -3ʹ and reverse, 5ʹ-CTCAACTGGTGTCGTGGA-3ʹ; and U6 forward, 5ʹ-CTCGCTTCGGCAGCACA-3ʹ and reverse, 5ʹ-AACGCTTCACGAATTTGCGT-3ʹ. 18srRNA and U6 were used as endogenous controls for SNHG16 and miR-342-3p expression, respectively. Fold-change in expression was calculated using the 2^-ΔΔCT^ method [12]. All experiments were repeated in independent triplicate.

### Cell proliferation, cycle, and apoptosis assay

Cell proliferation was evaluated using a CellTiter 96^®^ AQueous One Solution Cell Proliferation Assay (MTS assay; Promega, Madison, WI, USA). The absorbance was measured at 490 nm using a microplate reader (Bio-Rad, Hercules, CA, USA). Cell Cycle Detection Kit (Keygentec, Nanjing, China) was used to assessed the cell cycle. An Annexin V-FITC Apoptosis Detection Kit (Keygentec, Nanjing, China) was used to assessed cell apoptosis. The percentages of the cell population in different phases and cell apoptosis were assessed with flow cytometry (BD Biosciences, San Jose, CA, USA). All experiments were repeated in independent triplicate.

### Western blotting

Total protein samples from cells were prepared with RIPA lysis buffer with protease inhibitor (Beyotime, Shanghai, China). Equal quantities of denatured proteins (30 μg) were separated by SDS-PAGE and then transferred to polyvinylidene fluoride membranes. After blocking in Tris-buffered saline containing 0.1% Tween-20 (TBST) with 5% skim milk at room temperature for 2 h, each membrane was washed with TBST three times and incubated overnight at 4 °C with diluted primary antibodies: anti-Cyclin D1 antibody (ab134175, 1/500), anti-total-Caspase-3 antibody (ab4051, 1/1000), anti-Cleaved-Caspase-3 (ab2302, 1:500), anti-total-Caspase-9 antibody (ab32539, 1/1000), anti-FOXO3A (ab109629, 1:1000), anti-Bax (ab32503, 1:5000), anti-Bcl-2 (ab32124, 1:1000), anti-Cleaved Caspase-9 (ab2324, 1:100), anti- Phosphoinositide 3-kinase (PI3K) antibody (ab32089, 1/1000); anti-p-AKT antibody (ab8805, 1/500); anti-AKT antibody (ab16789, 1/1000), and anti-glyceraldehyde 3-phosphate dehydrogenase (GAPDH) antibody (ab181602, 1/2000). After incubation, membranes were washed with TBST three times, then incubated with horseradish peroxidase (HRP)-labeled secondary antibody (ab205718, 1/3000) for 2 h at room temperature and then washed with TBST three times. Finally, the proteins were quantified using enhanced chemiluminescence (Keygentec) and ChemiDoc™ XRS systems (Bio-Rad).

### Luciferase reporter assays

StarBase 3.0 software was used to predict miRNAs that targeted SNHG16. There are two miR-342-3p binding sites in the region of SNHG16. Wild-type SNHG16 (WT-SNHG16) containing putative miR-342-3p binding sites and SNHG16 containing mutated binding sites (MUT-SNHG16) (two miR-342-3p binding sites) were synthesized and then cloned into the luciferase reporter vector psi-CHECK-2 (Promega, Wisconsin, WI, USA). For luciferase reporter assays, HEK293 cells were co-transfected with luciferase reporter plasmids and miR-342-3p mimics, miR-342-3p inhibitor, or a negative control miRNA using Lipofectamine 2000. At 48 h post-transfection, cells were collected and relative luciferase activity was assessed using a Dual-Luciferase Reporter Assay System (Promega) according to the manufacturer’s instructions. The relative luciferase activity was normalized with Renilla luciferase activity. All experiments were repeated in independent triplicate.

### Statistical analysis

Statistical analyses were performed using SPSS 19.0 statistical software (IBM Inc., Chicago, IL, USA). Data are presented as mean ± standard deviation (SD). Differences were analyzed with *t*-test or one-way ANOVA. A *P*-value < 0.05 was regarded as statistically significant.

## Results

### SNHG16 is significantly up-regulated in MM samples and MM cells

**Fig. 1:**
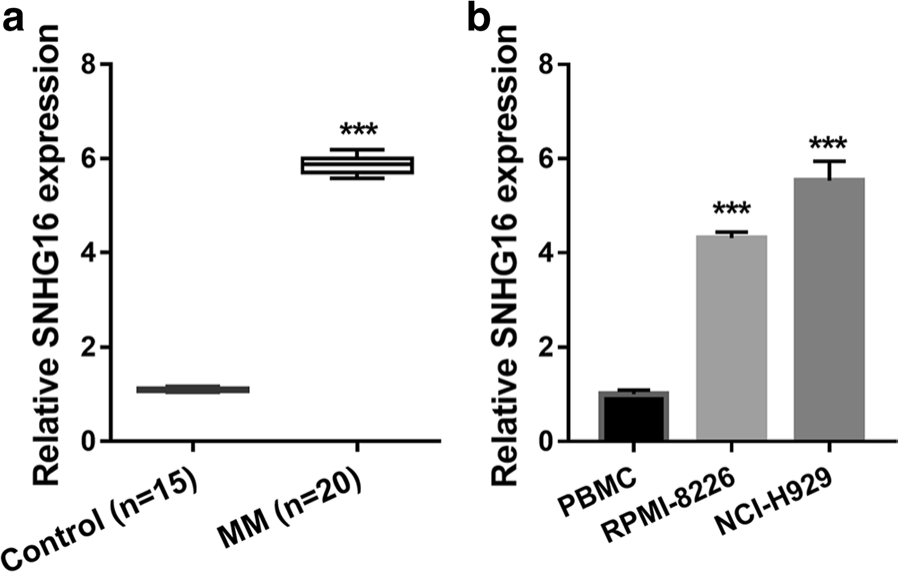
SNHG16 is significantly up-regulated in MM samples and MM cells. **a** Expression level of SNHG16 in MM samples were measured by qRT-PCR. **b** Additionally, SNHG16 expression in MM cell (RPMI-8226 and NCI-H929) and PBMC were measured by qRT-PCR at 24 h after cultured. ***P < 0.001

First, we found that SNHG16 expression was significantly up-regulated in MM patients compared with that in controls (normal marrow tissue) (Fig. 1a). Additionally, SNHG16 expression was significantly up-regulated in MM cell (RPMI-8226 and NCI-H929) compared with that in PBMC (Fig. 1b). The result suggested that SNHG16 might be involved in the progression of MM.

### Knockdown of SNHG16 suppresses cell proliferation in MM cells

**Fig. 2:**
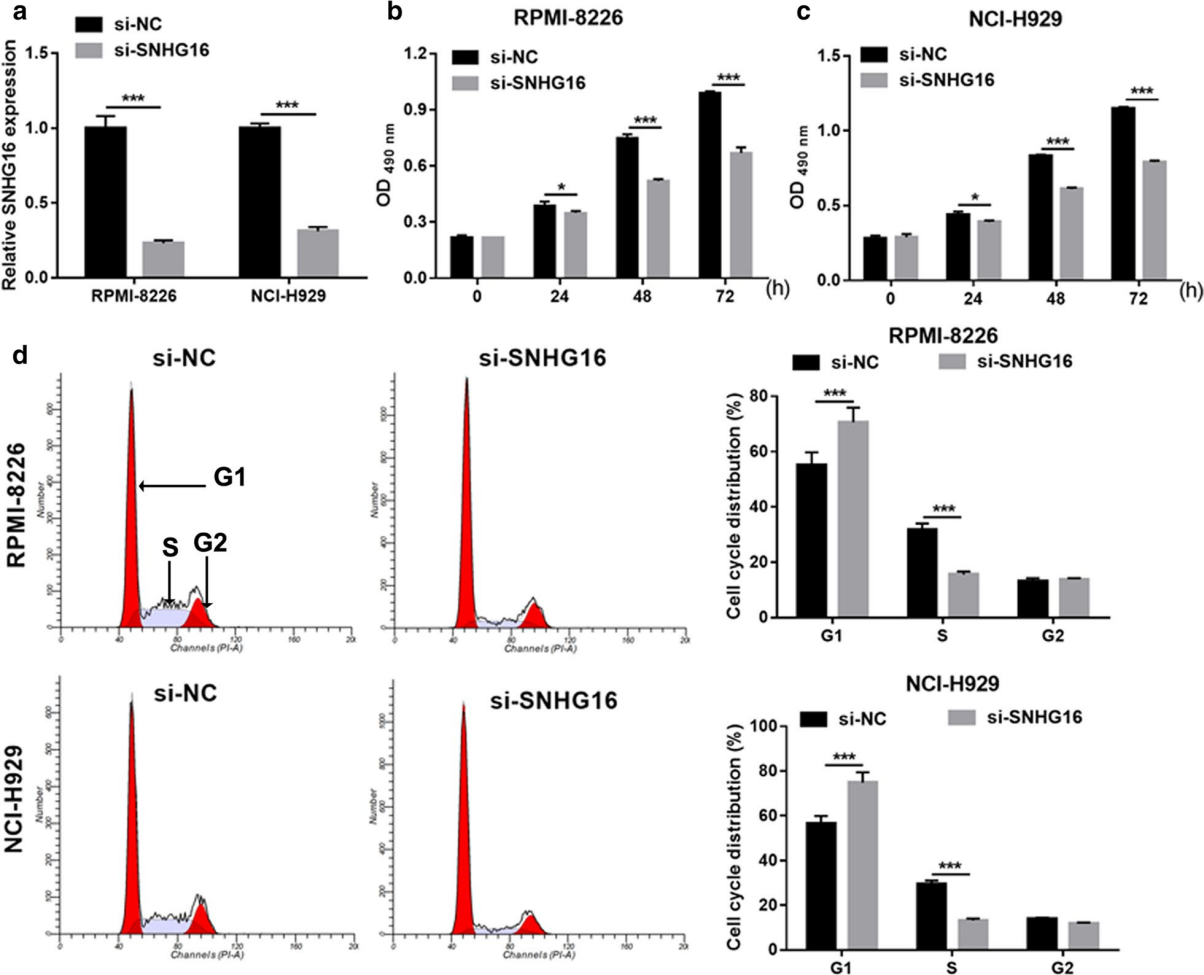
Knockdown of SNHG16 suppresses MM cell proliferation. **a** SNHG16 expression level in RPMI-8226 and NCI-H929 cells were measured using qRT-PCR at 48 h after transfected. **b**, **c** Cell proliferation of RPMI-8226 and NCI-H929 cells were examined by the MTS assay at 24 h, 48 h, and 72 h after transfected. **d** Cell cycle of RPMI-8226 and NCI-H929 cells were assessed by flow cytometry at 48 h after transfected. Left images: Represented image of cell cycle analyzed by flow cytometry. Frist red peak represented G1 phase. Grey peak represented S phase. Second red peak represented G2 phase. Right histograms: The bar graph represents the distribution of G1, S, and G2 phase per group. *P < 0.05, **P < 0.01, ***P < 0.001

**Fig. 3:**
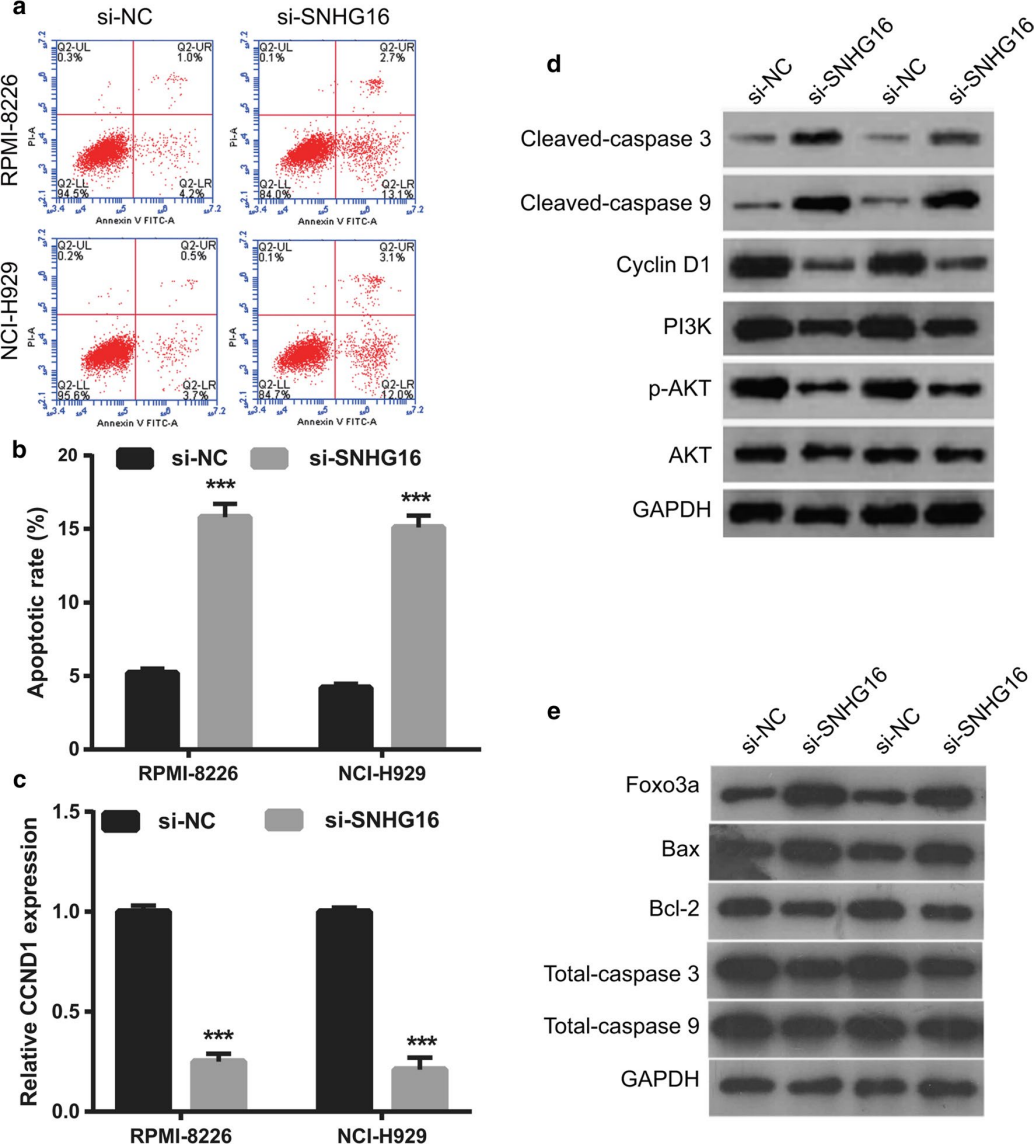
Knockdown of SNHG16 promoted MM cell apoptosis and regulation apoptosis and cell cycle-related proteins expression. **a** Represented image of apoptosis. Cell apoptosis of RPMI-8226 and NCI-H929 cells were assessed by flow cytometry at 48 h after transfected. **b** The bar graph represents the total apoptotic rate. The total apoptosis includes early apoptotic rate (LR) and late apoptotic rate (UR). ***P < 0.001. **c** The CCND1 expression was measured by qRT-PCR at 48 h after transfected. **d**, **e** The expression of Cleaved-Caspase-3/9, Cyclin D1, PI3K, p-AKT, AKT, Foxo3a, Bax, Bcl-2, and total Caspase-3/9 proteins in RPMI-8226 and NCI-H929 cells were measured by western blot at 48 h after transfected

To investigate the biological function of SNHG16 in MM, SNHG16 was knocked-down in RPMI-8226 and NCI-H929 cells by transfection with si-SNHG16 (Fig. 2a). SNHG16 knockdown significantly suppressed cell proliferation (Fig. 2b, c), arrested cell cycle transition from the G1 to S phase (Fig. 2d), and promoted cell apoptosis (Fig. 3a, b) both in RPMI-8226 and NCI-H929 cells compared with corresponding negative control (si-NC). Whereas, we found that PBMCs proliferation had no significant change between si-SNHG16 and si-NC groups (Additional file 1: Fig. S1). Additionally, SNHG16 knockdown markedly promoted the expression of cleaved-Caspase-3, cleaved-Caspase-9, Foxo3a, and Bax, markedly inhibited the expression of *CCND1*, Cyclin D1, PI3K, p-AKT, and Bcl-2, and had no effect on the expression of AKT and total caspase 3/9 in RPMI-8226 and NCI-H929 cells (Fig. 3c–e).

### SNHG16 directly interacts with miR-342-3p

**Fig. 4:**
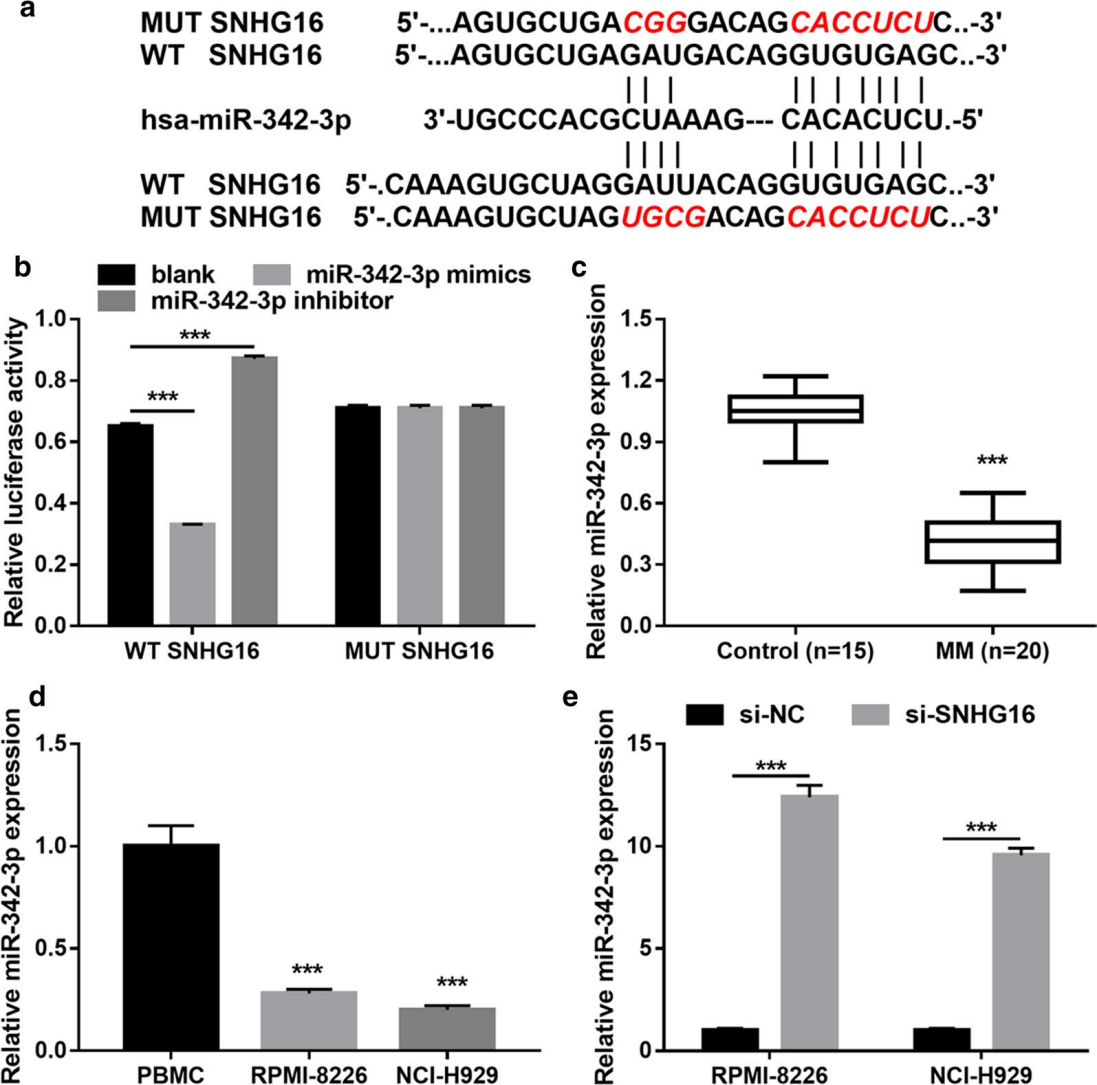
SNHG16 directly interacts with miR-342-3p. **a** Predicted miR-342-3p binding sites in the region of SNHG16 and the corresponding mutant sequence. **b** Interaction between miR-342-3p and SNHG16 assessed by luciferase reporter assays. **c** miR-342-3p expression level in MM samples were measured by qRT-PCR. **d** miR-342-3p expression level in MM cell (RPMI-8226 and NCI-H929) and PBMC were measured by qRT-PCR at 24 h after cultured. **e** MiR-342-3p expression level in RPMI-8226 and NCI-H929 cells were determined by RT-qPCR at 48 h after transfected. ***P < 0.001

To further investigate the molecular mechanism of SNHG16 on MM cell proliferation and apoptosis, potential target miRNAs were predicted using StarBase 3.0 online bioinformatics software. Two miR-342-3p binding sites in the region of SNHG16 were predicted (Fig. 4a). To confirm this predication, luciferase reporter assay was performed. The result revealed that miR-342-3p mimics markedly decreased the relative luciferase activity of the WT-SNHG16, while miR-342-3p inhibitor markedly increased the relative luciferase activity of the WT-SNHG16, but both had no effect on MUT-SNHG16 (Fig. 4b). In addition, miR-342-3p expression was significantly inhibited in MM tissues and RPMI-8226 and NCI-H929 cells (Fig. 4c, d). SNHG16 knockdown significantly increased miR-342-3p expression both in RPMI-8226 and NCI-H929 cells (Fig. 4e). These data indicated the direct interaction of SNHG16 with miR-342-3p in MM.

### Overexpression miR-342-3p suppresses MM cell proliferation in MM cells

**Fig. 5:**
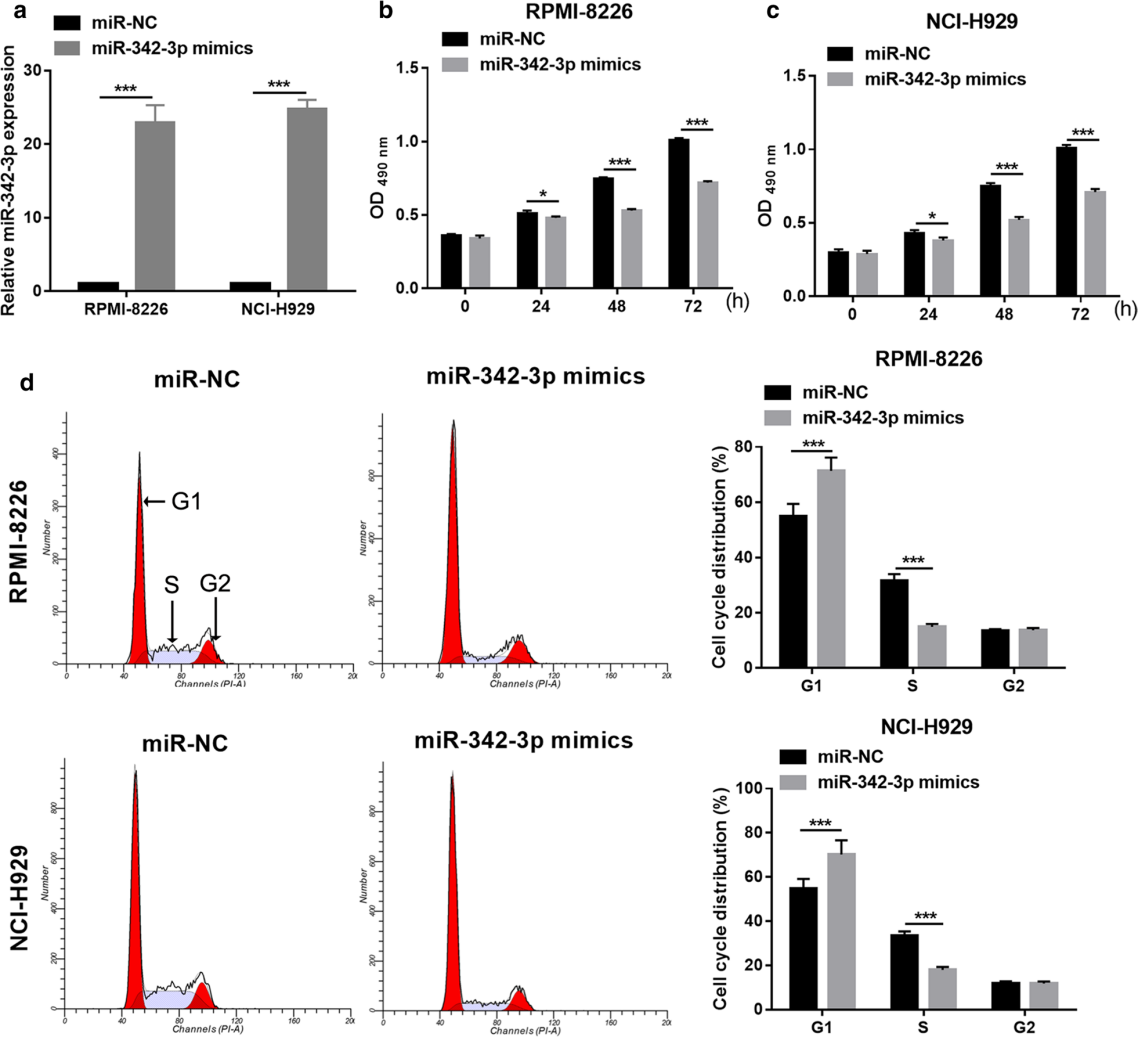
Overexpression miR-342-3p suppresses MM cell proliferation. **a** miR-342-3p expression level in RPMI-8226 and NCI-H929 cells were measured by qRT-PCR at 48 h after transfected. **b**, **c** Cell proliferation of RPMI-8226 and NCI-H929 cells were examined by MTS assay at 24 h, 48 h, and 72 h after transfected. **d** Cell cycle of RPMI-8226 and NCI-H929 cells were assessed by flow cytometry. Left images: represented image of cell cycle analyzed by flow cytometry. Frist red peak represented G1 phase. Grey peak represented S phase. Second red peak represented G2 phase. Right histograms: the bar graph represents the distribution of G1, S, and G2 phase per group. *P < 0.05, **P < 0.01, ***P < 0.001

**Fig. 6:**
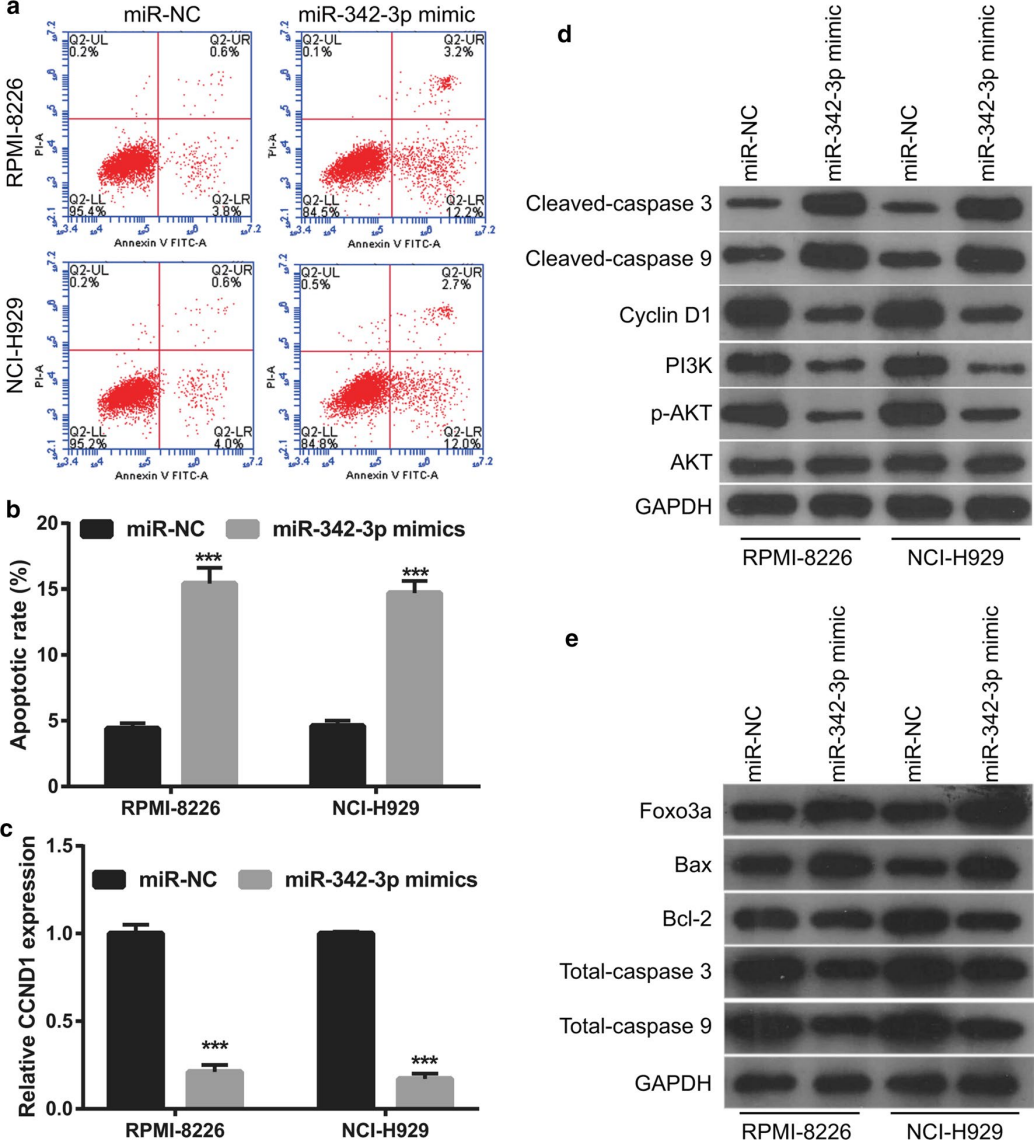
MiR-342-3p on expression promoted MM cell apoptosis and regulation apoptosis and cell cycle-related proteins expression. **a** Represented image of apoptosis. Cell apoptosis of RPMI-8226 and NCI-H929 cells were assessed by flow cytometry at 48 h after transfected. **b** The bar graph represents the total apoptotic rate. The total apoptosis includes early apoptotic rate (LR) and late apoptotic rate (UR). ***P < 0.001. **c** The CCND1 expression was measured by qRT-PCR at 48 h after transfected. **d**, **e** The expression of Cleaved-Caspase-3, Cleaved-Caspase-9, Cyclin D1, PI3K, p-AKT, AKT, Foxo3a, Bax, Bcl-2, and total Caspase-3/9 proteins in RPMI-8226 and NCI-H929 cells was measured by western blot at 48 h after transfection with miR-342-3p mimics or miR-NC

Next, we first overexpressed miR-342-3p in RPMI-8226 and NCI-H929 cells by transfection with miR-342-3p mimics. qRT-PCR revealed the significant overexpression of miR-342-3p in RPMI-8226 and NCI-H929 cells after transfection with miR-342-3p mimics compared with that transfected with miR-NC (Fig. 5a). Overexpression miR-342-3p significantly suppressed cell proliferation, arrested cell cycle transition from the G1 to S phase (Fig. 5b–d), and promoted cell apoptosis (Fig. 6a, b) in RPMI-8226 and NCI-H929 cells. The overexpression of miR-342-3p markedly promoted the expression levels of cleaved-Caspase-3, cleaved-Caspase-9, Foxo3a, and Bax, markedly inhibited the expression of *CCND1*, Cyclin D1, PI3K, p-AKT, and Bcl-2, and had no effect on the expression levels of AKT and total caspase 3/9 in RPMI-8226 and NCI-H929 cells (Fig. 6c–e).

### MiR-342-3p inhibition attenuates the effect of SNHG16 knockdown on cell proliferation in MM cells

**Fig. 7:**
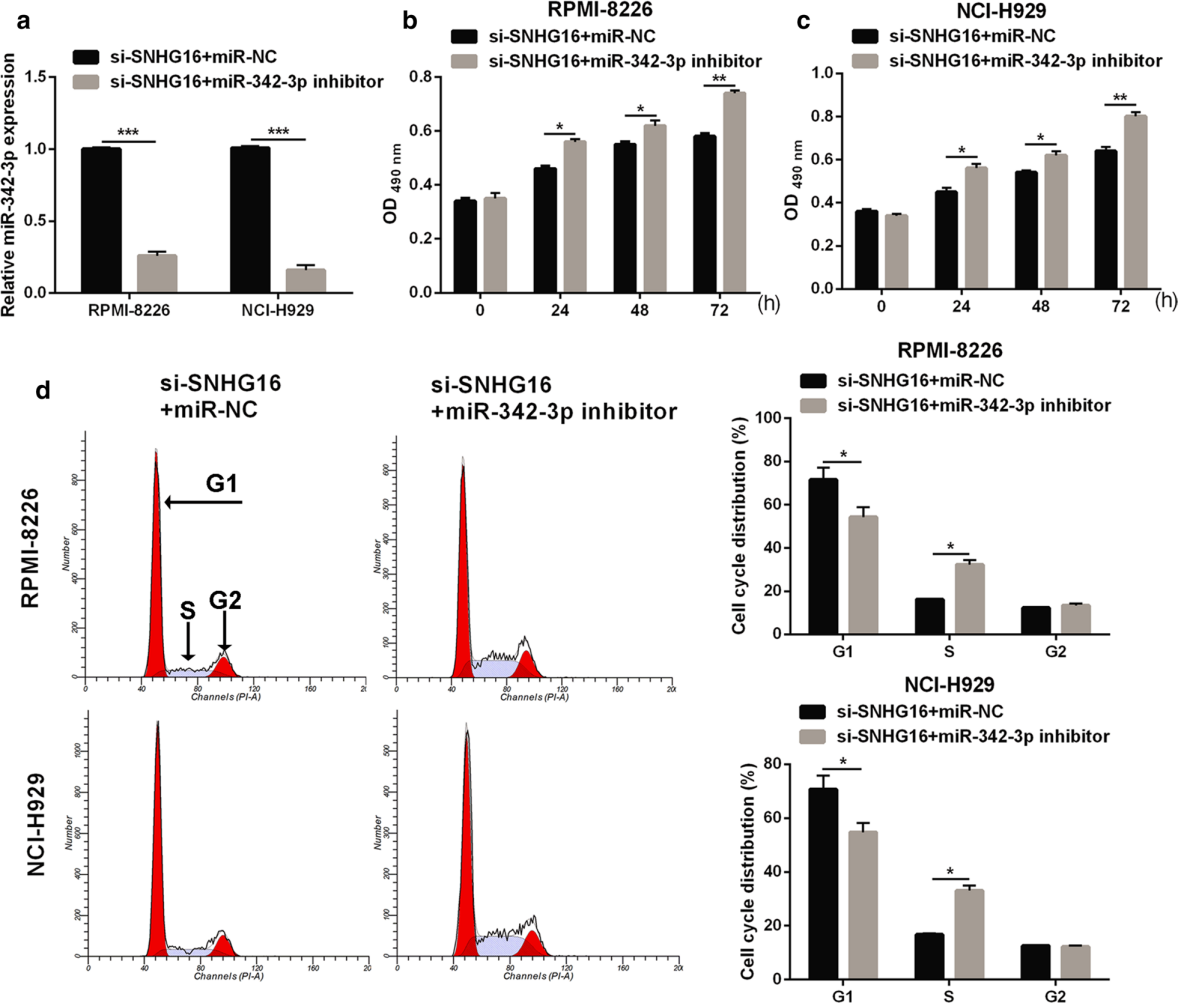
MiR-342-3p inhibitor attenuates the effect of SNHG16 knockdown on cell proliferation of MM cells **a** MiR-342-3p expression level in RPMI-8226 and NCI-H929 cells were measured by qRT-PCR at 48 h after transfected. **b**, **c** Cell proliferation of RPMI-8226 and NCI-H929 cells were examined by the MTS assay at 24 h, 48 h, and 72 h after transfected. **d** Cell cycle of RPMI-8226 and NCI-H929 cells were assessed by flow cytometry at 48 h after transfected. Left images: represented image of cell cycle analyzed by flow cytometry. Frist red peak represented G1 phase. Grey peak represented S phase. Second red peak represented G2 phase. Right histograms: the bar graph represents the distribution of G1, S, and G2 phase per group. *P < 0.05, **P < 0.01, ***P < 0.001

**Fig. 8:**
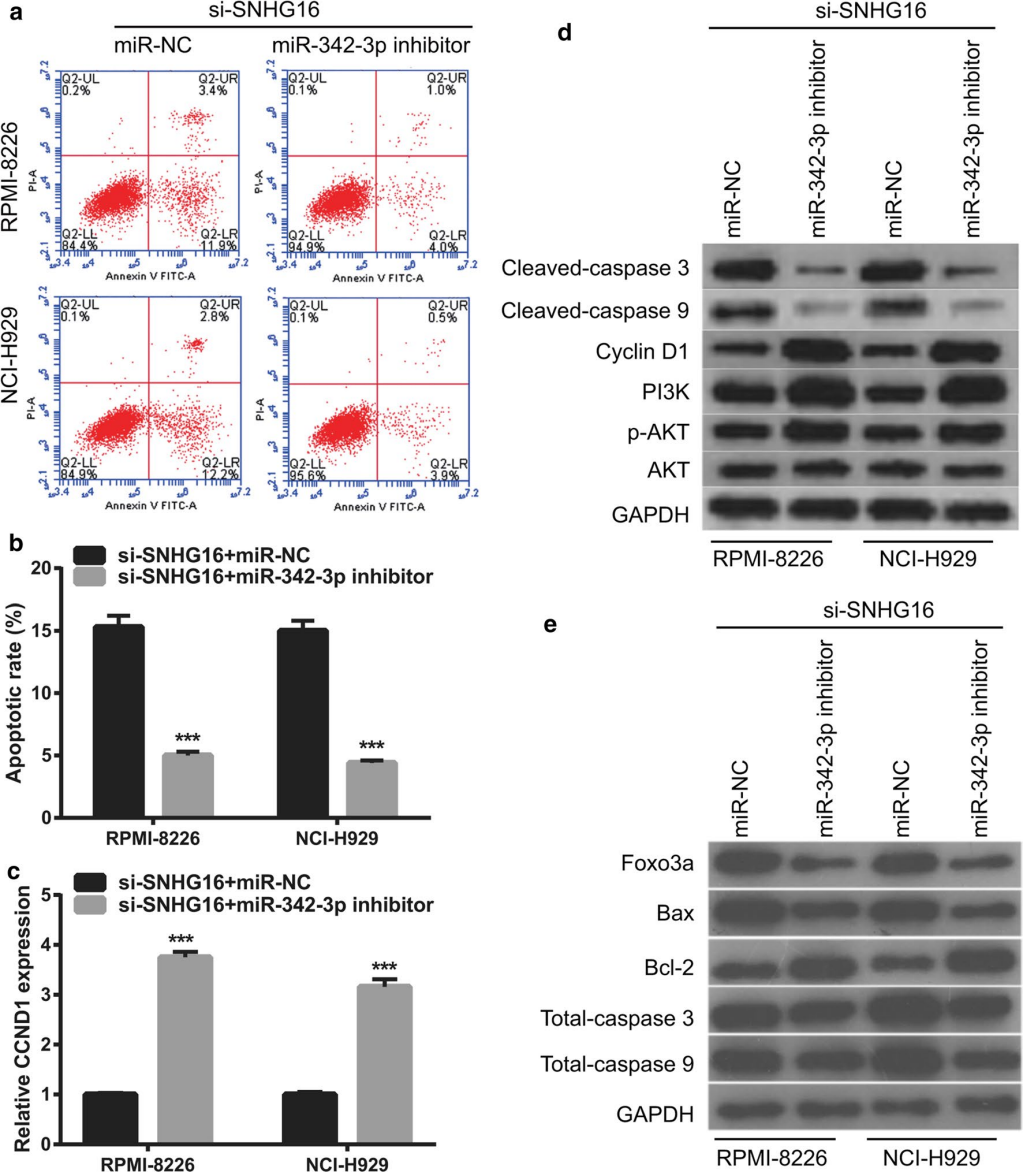
MiR-342-3p inhibitor attenuates the effect of SNHG16 knockdown on cell apoptosis of MM cells and apoptosis and cell cycle-related proteins expression. **a** Represented image of apoptosis. Apoptosis of RPMI-8226 and NCI-H929 cells were assessed by flow cytometry at 48 h after transfected. **b** The bar graph represents the total apoptotic rate. The total apoptosis includes early apoptotic rate (LR) and late apoptotic rate (UR). ***P < 0.001**. c** The CCND1 expression was measured by qRT-PCR at 48 h after transfected. **d**, **e** The expression of Cleaved-Caspase-3, Cleaved-Caspase-9, Cyclin D1, PI3K, p-AKT, -AKT, Foxo3a, Bax, Bcl-2, and total Caspase-3/9 proteins in RPMI-8226 and NCI-H929 cells were measured by western blot at 48 h after transfected

To further verify that SNHG16 exerts its biological role by regulating miR-342-3p, a rescue experiment was performed by inhibiting miR-342-3p expression in RPMI-8226 and NCI-H929 cells with SNHG16 knockdown. The expression of miR-342-3p was significantly decreased in RPMI-8226 and NCI-H929 cells transfected with si-SNHG16 and miR-342-3p inhibitor (Fig. 7a). Moreover, miR-342-3p inhibition promoted cell proliferation (Fig. 7b, c), enhanced cell cycle transition from the G1 to S phase (Fig. 7d), and suppressed apoptosis (Fig. 8a, b) in RPMI-8226 and NCI-H929 cells transfected with si-SNHG16. Additionally, the miR-342-3p inhibitor elevated the expression levels of *CCND1*, Cyclin D1, PI3K, p-AKT, and Bcl-2, decreased expression of cleaved-Caspase-3, cleaved-Caspase-9, Foxo3a, and Bax, and had no effect on AKT and total caspase 3/9 expression in RPMI-8226 and NCI-H929 cells transfected with si-SNHG16 (Fig. 8c–e).

## Discussion

Abnormal expression of lncRNAs contribute to the tumorigenesis and development of MM, and can act as valuable diagnosis markers and attractive therapeutic targets for MM [13, 14]. In the present study, SNHG16 expression was markedly increased in tissue from MM patients. Furthermore, SNHG16 knockdown suppressed cell proliferation, induced cell cycle arrest, and promoted apoptosis of MM cells by sponging miR-342-3p. Thus, SNHG16 may serve as a novel biomarker for MM.

SNHG16 is reportedly increased and acts as a tumor promoting role in various cancers. For example, Xie et al. [15] found that SNHG16 was up-regulated in hepatocellular carcinoma tissues and cell lines, and that SNHG16 promoted hepatocellular carcinoma cell proliferation, invasion and tumorigenesis. Liu et al. [7] reported that SNHG16 was increased in pancreatic cancer tissues, SNHG16 knockdown suppressed cell proliferation and metastasis. Presently, SNHG16 expression was markedly up-regulated in samples from MM patients and in cell lines. Function assays showed that SNHG16 knockdown suppressed cell proliferation, arrested cell cycle transition from G1 to S phase, and promoted cell apoptosis in MM cells, similar with the roles of SNHG16 in hepatocellular carcinoma and pancreatic cancer. The PI3K/AKT pathway has been demonstrated to play a central role in cell growth and proliferation, and is associated with various cancers [16]. To further investigate the regulatory mechanism of SNHG7 on MM cell proliferation at the protein level, we focused on the effect of SNHG16 knockdown on the expression of relevant proteins. SNHG16 knockdown promoted cleaved-Caspase-3, cleaved-Caspase-9 expression, Foxo3a, and Bax, markedly inhibited *CCND1*, Bcl-2, Cyclin D1, PI3K, and p-AKT expression in MM cells. Thus, SNHG16 might acted as an oncogene and promote cell proliferation and apoptosis by regulating PI3K/AKT pathway in MM cells.

Recent evidence has indicated that lncRNAs can function as molecular sponges for miRNAs to regulate the expression and function of target miRNAs [17]. SNHG16 has been reported to function as a molecular sponge for multiple miRNAs in cancers, such as miR-98-5p [18], miR-135a [8], and miR-373 [19]. We investigated the molecule mechanism of SNHG16 regulation in the progression of MM by using bioinformatics analysis to predict putative binding miRNAs. Researchers have reported miR-342-3p as tumor suppressors in various cancers. For instance, overexpression of miR-342-3p inhibits cell proliferation in hepatocellular carcinoma through the inhibition of insulin-like growth factor 1-mediated Warburg effect [20]. miR-342-3p inhibits non-small cell lung cancer cell growth and migration by targeting Anterior Gradient 2 [10]. Here, we predicted that SNHG16 shares two complementary binding sites for miR-342-3p. Furthermore, SNHG16 functioned as a molecular sponge for miR-342-3p and SNHG16 knockdown significantly increased miR-342-3p expression in MM cells. Importantly, we further demonstrated that the overexpression of miR-342-3p has similar effects with SNHG16 knockdown on MM cell proliferation, cycle, apoptosis, and related protein expression, while repression of miR-342-3p could rescue the effect of SNHG16 knockdown on MM cell proliferation, cycle arrest, apoptosis, and related protein expression. Taken together, these findings indicate that SNHG16 has an oncogenic role by sponging miR-342-3p in MM cells.

## Conclusion

SNHG16 was up-regulated in MM tissues, knockdown of SNHG16 suppresses cell proliferation, induced cycle arrest, and promoted apoptosis of MM cells by sponging miR-342-3p. These findings may contribute to a better understanding the role of SNHG16 in MM pathogenesis. SNHG16 might be a potential target for treatment of MM.

## Supplementary information

**Additional file 1: Fig. S1.** Knockdown of SNHG16 had no effect on PBMCs proliferation. **a** SNHG16 expression level in PBMCs cell was measured using qRT-PCR at 48 h after transfected. **b** Cell proliferation of PBMCs cells was examined by the MTS assay at 24 h, 48 h, and 72 h after transfected.

## Data Availability

The data supporting the conclusions of this paper are included within the article.
