# Supplementary figures and images for: Knockdown of lncRNA SNHG16 suppresses multiple myeloma cell proliferation by sponging miR-342-3p
Source: Cancer Cell Int. 2020 Feb 3;20:38. doi: 10.1186/s12935-020-1118-1 (PMC6998159; doi:10.1186/s12935-020-1118-1)

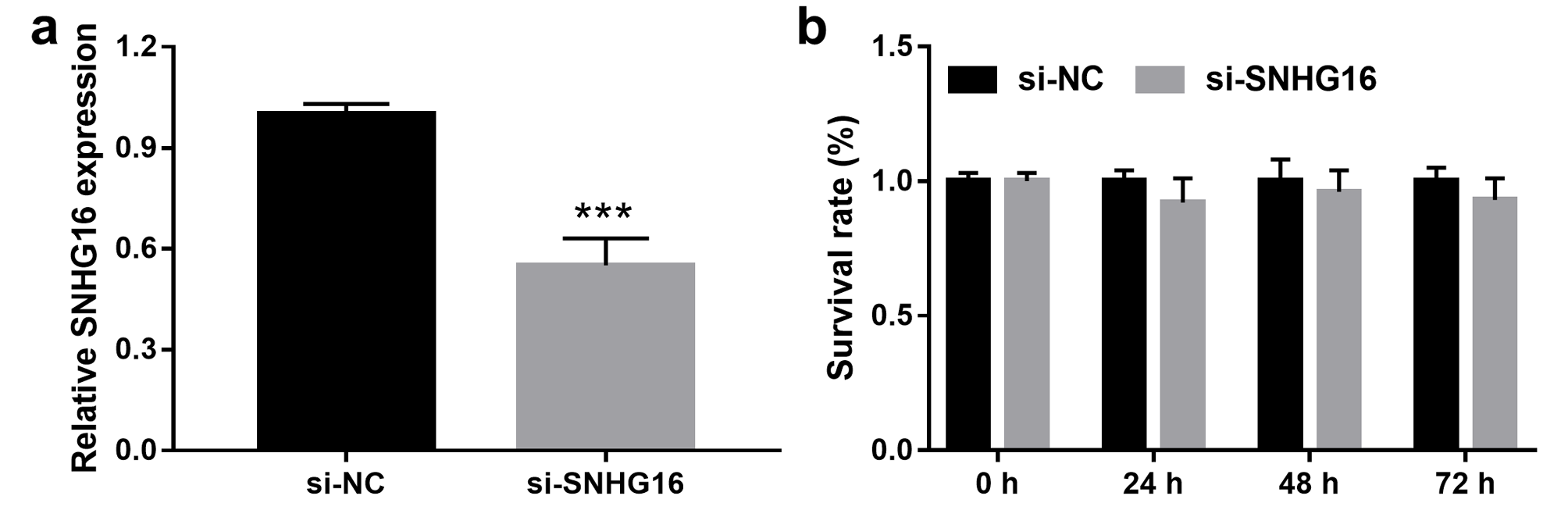

Supplement: Supplementary file 1 — Additional file 1: Fig. S1. Knockdown of SNHG16 had no effect on PBMCs proliferation. a SNHG16 expression level in PBMCs cell was measured using qRT-PCR at 48 h after transfected. b Cell proliferation of PBMCs cells was examined by the MTS assay at 24 h, 48 h, and 72 h after transfected. [file 12935_2020_1118_MOESM1_ESM.tif]
